# A Simple-to-Use Web-Based Calculator for Survival Prediction in Acute Respiratory Distress Syndrome

**DOI:** 10.3389/fmed.2021.604694

**Published:** 2021-02-16

**Authors:** Yong Liu, Jian Liu, Liang Huang

**Affiliations:** Department of Emergency, The First Affiliated Hospital of Nanchang University, Nanchang, China

**Keywords:** acute respiratory distress syndrome, LASSO regression, nomogram, model, survival

## Abstract

**Background:** The aim of this study was to construct and validate a simple-to-use model to predict the survival of patients with acute respiratory distress syndrome.

**Methods:** A total of 197 patients with acute respiratory distress syndrome were selected from the Dryad Digital Repository. All eligible individuals were randomly stratified into the training set (*n*=133) and the validation set (*n*=64) as 2: 1 ratio. LASSO regression analysis was used to select the optimal predictors, and receiver operating characteristic and calibration curves were used to evaluate accuracy and discrimination of the model. Clinical usefulness of the model was also assessed using decision curve analysis and Kaplan-Meier analysis.

**Results:** Age, albumin, platelet count, PaO_2_/FiO_2_, lactate dehydrogenase, high-resolution computed tomography score, and etiology were identified as independent prognostic factors based on LASSO regression analysis; these factors were integrated for the construction of the nomogram. Results of calibration plots, decision curve analysis, and receiver operating characteristic analysis showed that this model has good predictive ability of patient survival in acute respiratory distress syndrome. Moreover, a significant difference in the 28-day survival was shown between the patients stratified into different risk groups (*P* < 0.001). For convenient application, we also established a web-based calculator (https://huangl.shinyapps.io/ARDSprognosis/).

**Conclusions:** We satisfactorily constructed a simple-to-use model based on seven relevant factors to predict survival and prognosis of patients with acute respiratory distress syndrome. This model can aid personalized treatment and clinical decision-making.

## Introduction

Acute respiratory distress syndrome (ARDS) is a clinically and pathophysiologically complex syndrome characterized by rapid progression and devastating hypoxemic respiratory failure (1). Many risk factors, such as sepsis, pneumonia, pancreatitis, and major trauma, are associated with the development of ARDS (2). Although there has been some progress in ARDS treatment in the last several decades, the prognosis of patients with ARDS are still not satisfactory. The in-hospital mortality rate of ARDS patients remains between 34 and 60% (3). At present, the treatment of ARDS predominantly includes mechanical ventilation therapy (4). Therefore, identification of novel and effective treatment strategies is crucial for patients with ARDS. Moreover, a simple-to-use clinical prediction model is also required to provide adequate care to patients with ARDS.

The severity of ARDS is often assessed using the PaO_2_/FiO_2_ ratio, although this variable has a low-to-moderate prognostic value (5). Recently, several biomarkers including inflammation cytokines, epithelial or endothelial damage, and coagulation have been established to evaluated prognosis and therapeutic response of patients with ARDS. For example, a meta-analysis reported that elevated plasma levels of angiopoietin-2 strongly correlate with diagnosis and mortality in populations at high risk of ARDS (6). Moreover, various clinical biomarkers including lung inflammatory mediators (soluble suppression of tumorigenicity-2 and interleukin-6) (7) and products of epithelial and endothelial injury (the soluble form of the receptor for advanced glycation end products) (8, 9) were developed to monitor pathophysiologic changes and outcomes of ARDS. Unfortunately, although several lung-specific biomarkers have been validated to assess ARDS; however, none of them have been applied into clinical practice. Currently, there is no favorable prognosis prediction model for ARDS.

Nomograms (visualized graphs of a predictive model) are widely applied for prognosis and prediction of various diseases (10, 11). To date, no nomogram and corresponding web-based calculator has been developed to predict the prognosis of ARDS patients. Therefore, a refined model is needed to predict the prognosis of ARDS and guide clinical treatment. In this study, we aimed to construct a web-based calculator to predict the 28-day survival of patients with ARDS using several clinical parameters that are routinely used and readily available. This simple-to-use calculator might serve as an early warning and prediction system for patients with ARDS.

## Methods

### Patients

A total of 197 patients with ARDS were extracted from the Dryad Digital Repository (http://www.datadryad.org/), which was shared by Anan et al. (12). All ARDS patients were diagnosed according to the Berlin definition (5). Patients with chronic interstitial lung disease (idiopathic pulmonary fibrosis), vasculitis or alveolar hemorrhage, hypersensitivity pneumonitis were excluded. All eligible patients were randomly stratified into two groups in a 2:1 ratio (training set and validation set, respectively). The extracted clinical data included age, gender, white cell count (WBC), C-reactive protein (CRP), lactate dehydrogenase (LDH), albumin (Alb), platelet count (PLT), PEEP, SOFA score, high-resolution computed tomography (HRCT) score, McCabe score, PaO2/FiO2, ARDS etiology, survival time, and survival status. Institutional ethical approval was not necessary because all the data were obtained from an online database.

### Development of the Nomogram

To obtain the subset of predictors, the LASSO regression analysis was used to select the optimal predictors from the risk factors in the training cohort. The “glmnet” package was used to perform the LASSO regression analysis (13, 14). Finally, using the selected predictors from the LASSO regression, a nomogram was developed using the “rms,” “survival,” “foreign,” and “openxlsx” R packages (15–18). A dynamic web-based calculator was constructed using “DynNom” package (19).

### Validation of the Nomogram

To validate the constructed nomogram, the corresponding calibration map and receiver operating characteristic (ROC) analysis were performed in the training and validation sets to assess the prognostic accuracy of the nomogram by using the “rms,” “survival,” “foreign,” and “timeROC” R packages (20). In addition, decision curve analysis (DCA) was performed to quantify the clinical applicability of the nomogram.

### Statistical Analysis

The raw data were expressed as mean ± standard deviation when normally distributed, while expressed as median (interquartile range) when non-normally distributed. Differences between two groups were analyzed using chi-square tests for categorical variables and t-tests for continuous variables. The Kaplan–Meier method and the log-rank test were used to estimate survival. All statistical analyses were performed using R software (Version 3.6.2; http://www.Rproject.org). A two-sided *P*-value < 0.05 was considered to indicate statistical significance.

## Results

### Baseline Characteristics

In total, 197 eligible ARDS patients with integrated information were randomly stratified into two independent cohorts (training set, *n* = 133; validation set, *n* = 64). Patients' baseline clinical characteristics are shown in Table 1. A total of 123 male patients and 74 female patients were enrolled in this study. The average age of the patients was 73.94 ± 11.92 years. After 28 days of follow-up, 69 (35.0%) patients died during the entire study population.

**Table 1:** Baseline characteristics of included patients in training and validation sets.

**Characteristic**	**Entire cohort (*n* = 197)**	**Training set (*n* = 133)**	**Validation set (*n* = 64)**	***P*-value**
**Age, years**	73.94 ± 11.92	74.41 ± 11.95	72.97 ± 11.90	0.427
**Sex**				0.647
Female	74(37.6%)	48(36.1%)	26(40.6%)	
Male	123(62.4%)	85(63.9%)	38(59.4%)	
**Alb, g/Dl**	2.84 ± 0.58	2.81 ± 0.58	2.90 ± 0.59	0.317
**PLT, per mm**^**3**^	19.23 ± 10.56	19.18 ± 10.50	19.32 ± 10.75	0.927
**WBC, per mm**^**3**^	11010.66 ± 7255.91	10600.75 ± 7076.02	11862.50 ± 7602.22	0.254
**CRP, mg/dl**	17.42 ± 10.66	16.77 ± 10.83	18.77 ± 10.26	0.219
**SOFA score**	7.71 ± 3.47	8.09 ± 3.63	6.91 ± 2.98	0.024
**McCabe score**				0.474
1	174 (88.3%)	115 (86.5%)	59 (92.2%)	
2	11 (5.6%)	9 (6.8%)	2 (3.1%)	
3	12 (6.1%)	9 (6.8%)	3 (4.7%)	
**PaO2/FiO2**	116.11 ± 50.96	117.66 ± 50.57	112.89 ± 52.01	0.540
**LDH, IU/L**	390.57 ± 231.73	386.68 ± 199.63	398.64 ± 288.90	0.735
**HRCT score**	236.69 ± 66.70	233.46 ± 64.94	243.41 ± 70.27	0.328
**PEEP, cmH_2_O**	10.40 ± 5.23	10.14 ± 5.22	10.92 ± 5.25	0.329
**ARDS etiology**				0.036
DARDS	170(86.3%)	120(90.2%)	50(78.1%)	
Non-DARDS	27(13.7%)	13(9.8%)	14(21.9%)	
**Vital status**				0.212
Living	128(65.0%)	82(61.7%)	46(71.9%)	
Deceased	69(35.0%)	51(38.3%)	18(28.1%)	

*Alb, albumin; PLT, platelet count; WBC, white cell count; CRP, C reactive protein; SOFA, sequential organ failure assessment; LDH, lactate dehydrogenase; HRCT, high-resolution computed tomography; DARDS, drug-associated ARDS*.

### Construction of the Model

**Figure 1:**
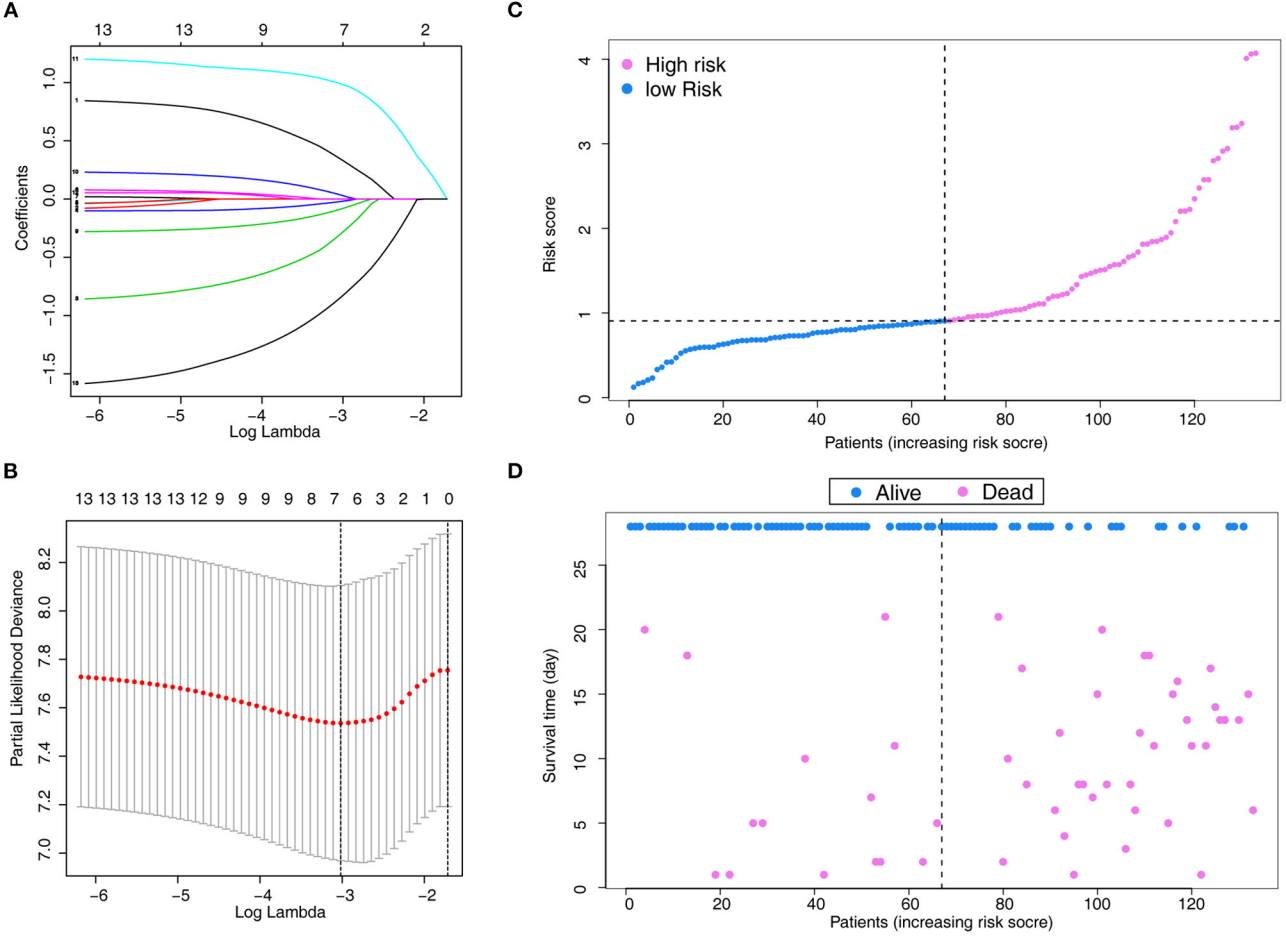
Parameter selection using LASSO regression. **(A)** LASSO coefficient profiles of the 13 features. A coefficient profile plot is produced against the log(λ) sequence. **(B)** Variables selected through LASSO with 10-fold cross-validation. **(C)** Distribution of the risk score. **(D)** Relationships between survival status and survival times of ARDS patients ranked by risk score. The black dotted line represents the optimum cut-off point dividing patients into low- and high-risk groups. LASSO, least absolute shrinkage and selection operator.

**Figure 2:**
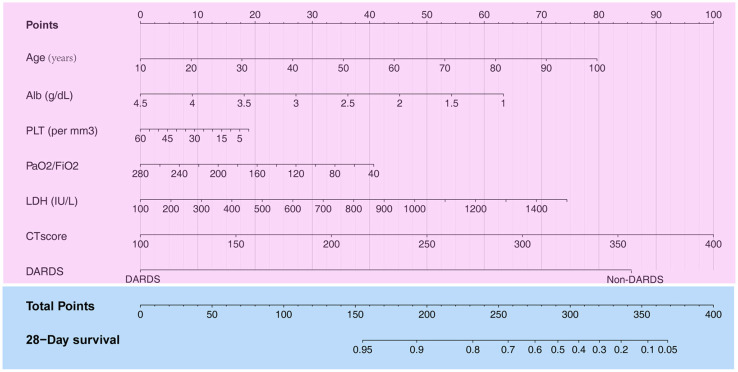
Construction of a nomogram with clinical indices to predict ARDS-related survival (based on the training set). The score for each value is assigned by drawing a line upward to the points line, and the sum of the seven scores is plotted on the Total points line. ARDS, acute respiratory distress syndrome.

**Figure 3:**
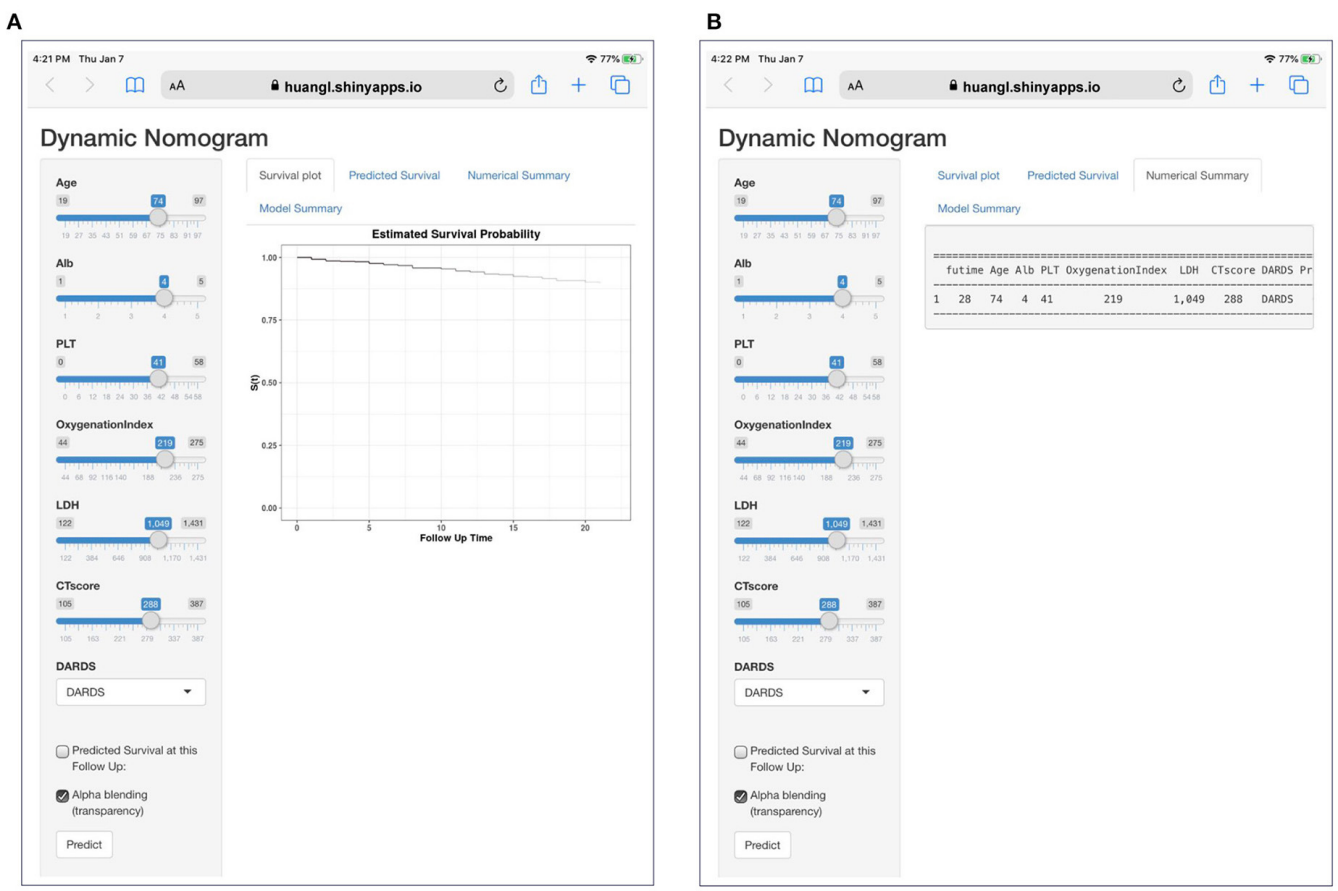
A dynamic web-based calculator to predict ARDS-related survival (https://huangl.shinyapps.io/ARDSprognosis/). **(A)** Web survival rate calculator. **(B)** 95% confidence interval of the web survival rate calculator.

A total of 13 parameters were used for LASSO regression, and seven parameters were selected as the optimal predictors by LASSO (Figures 1A,B). The seven retained variables were then used to construct the predictive model. The risk-score for each individual was calculated based on the model coefficients combined with the corresponding value of the identified seven clinical parameters. Thereafter, the patients were classified into low- and high-risk groups in both cohorts according to the median risk-score. Figures 1C,D show the risk-score distribution and the survival status of individual in the high- and low-risk cluster. The variables including Age, Alb, PLT, PaO_2_/FiO_2_, LDH, HRCT, and etiology were incorporated into the nomogram (Figure 2). To facilitate the clinical application of our findings, we developed a web-based calculator (https://huangl.shinyapps.io/ARDSprognosis/) to predict prognosis of ARDS patients according to the nomogram (Figures 3A,B). The estimated 28-day survival probabilities could be obtained by drawing a perpendicular line from the total point axis to the outcome axis.

### Performance of the Model

**Figure 4:**
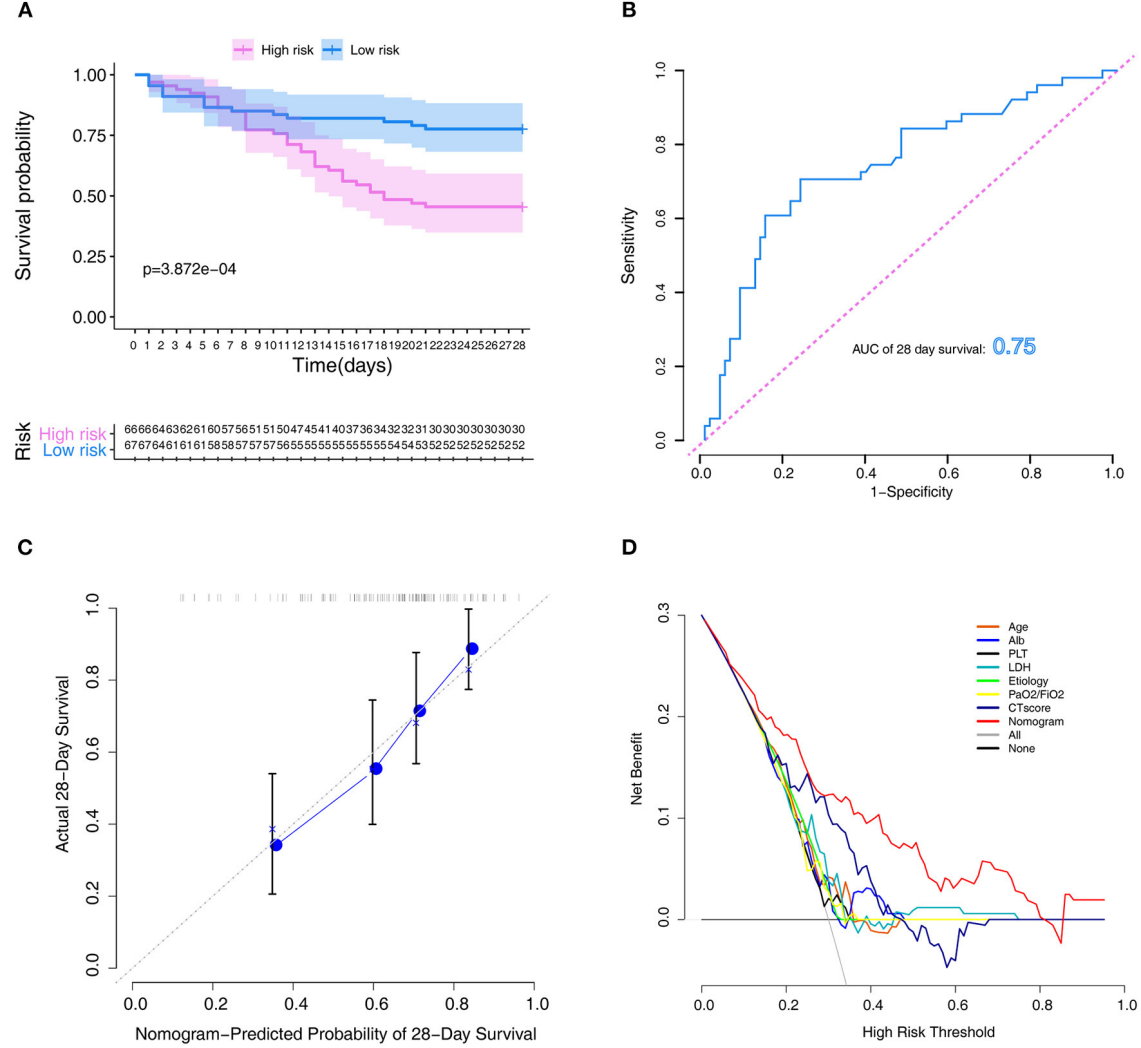
Assessment of the model in the training set. **(A)** Kaplan–Meier survival analysis between the high- and low-risk groups. **(B)** ROC curves of predictive models at 28 days. **(C)** Calibration plot for the training set that show the predicted and observed (with 95% confidence intervals) overall survival rates at 28 days. Model performance is shown by the plot, relative to the 45-degree line, which represents perfect prediction. **(D)** Decision curve of the model. The gray line represents the treat-all-patients scheme. The dotted line represents the treat-none scheme. The red line represents prediction nomogram scheme in training dataset. The X axis represents threshold probability. The Y axis represents net benefit. ROC, receiver operator characteristic.

The Kaplan–Meier survival curves revealed significantly poor overall survival in the high-risk group (*p* = 3.872e-04; Figure 4A). Thereafter, we performed ROC analysis to assess the discriminability of the model. The area under the ROC curve (AUC) indicative of the 28-day survival prediction was 0.75 (Figure 4B), which implied an efficacious performance of the model to predict prognosis. The calibration plots based on the training set showed that the model could accurately predict the 28-day survival (Figure 4C). The results of DCA also exhibited that the model could help clinicians to obtain maximum benefit when making clinical decisions (Figure 4D).

**Figure 5:**
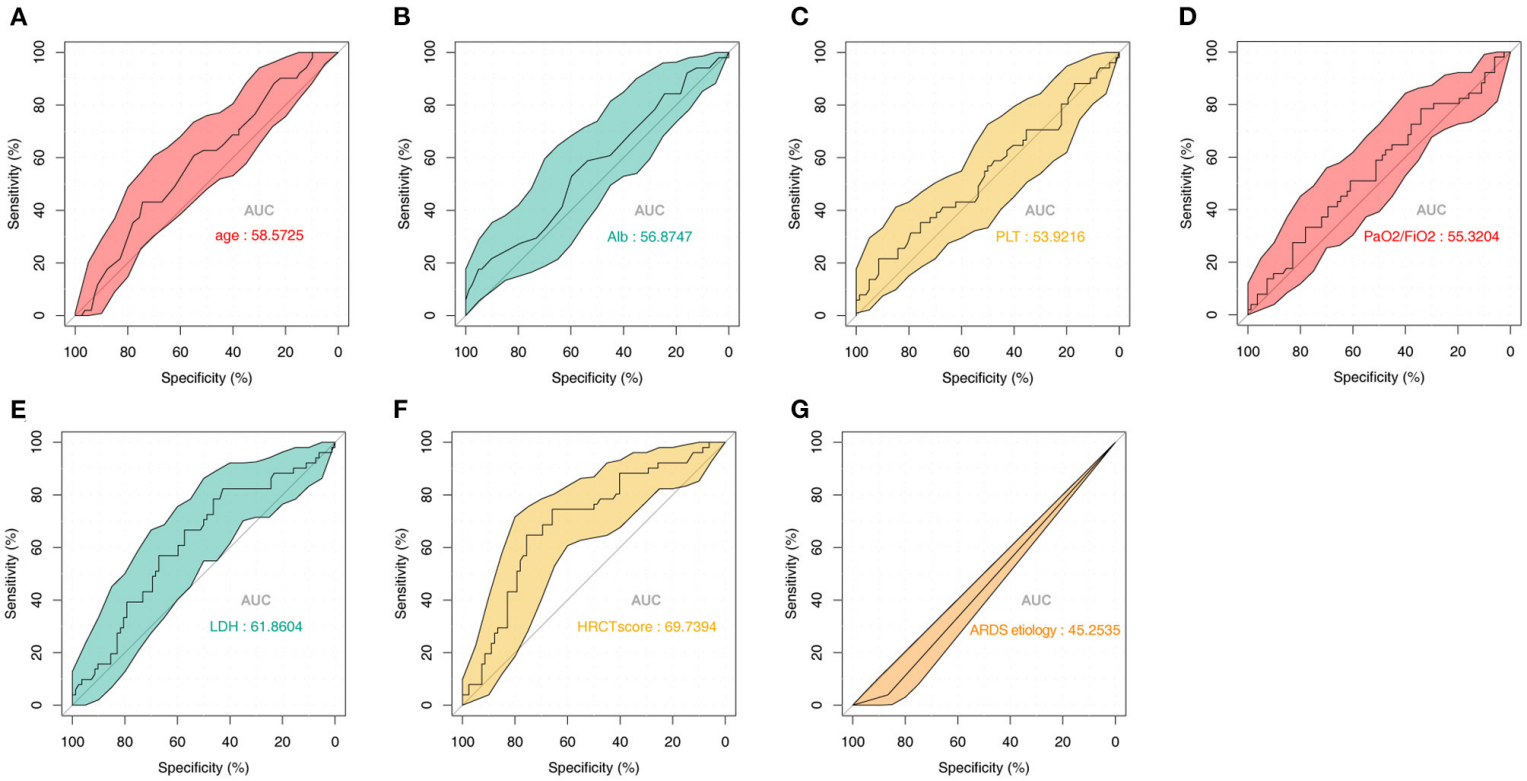
Predictive value of the seven parameters included in the model from the training set. ROC analysis of **(A)** Age, **(B)** Alb, **(C)** PLT, **(D)** PaO_2_/FiO_2_, **(E)** LDH, **(F)** HRCT score, and **(G)** ARDS etiology. ROC, receiver operator characteristic; Alb, albumin; PLT, platelet; LDH, lactate dehydrogenase; HRCT, high-resolution computed tomography; ARDS, acute respiratory distress syndrome.

To further study the predictive value of each parameter included in the model, we performed ROC analysis for each of them (Figures 5A–G). The AUC values of all parameters were lower than that of the complete nomogram model. These results demonstrated that the model had superior predictive performance and clinical value than any single factor.

### Performance Validation of the Model

**Figure 6:**
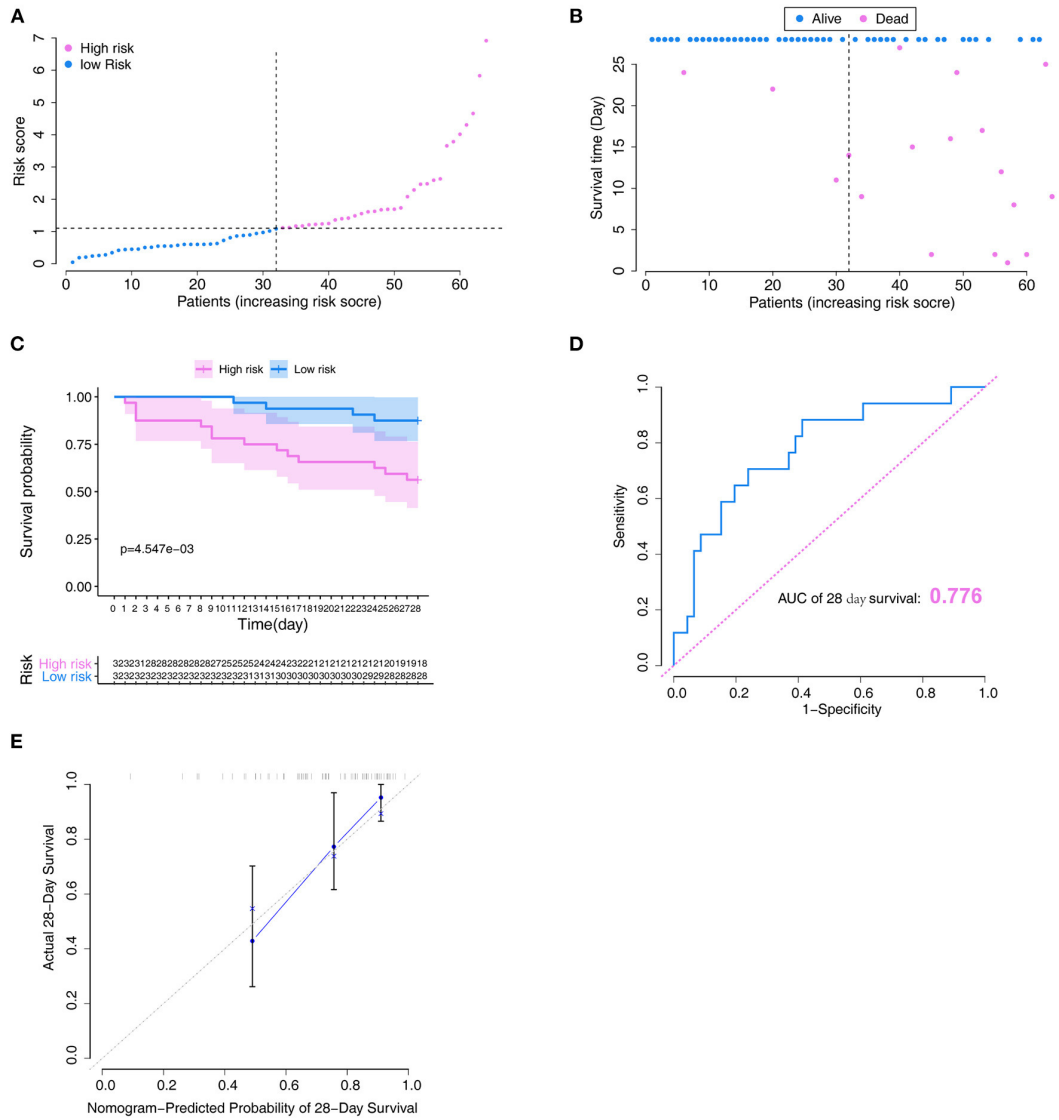
Verification of the model in the validation set. **(A)** Distribution of the risk score. **(B)** Relationships between survival status and survival times of ARDS patients ranked by risk score. The black dotted line represents the optimum cut-off point dividing patients into low- and high-risk groups. **(C)** Kaplan–Meier survival analysis between high- and low-risk groups. **(D)** ROC curves of predictive models at 28 days. **(E)** Calibration plot for the training set that show the predicted and observed (with 95% confidence intervals) overall survival rates at 28 days. Model performance is shown by the plot, relative to the 45-degree line, which represents perfect prediction. ROC, receiver operator characteristic.

To verify the reliability of the constructed novel model, risk-scores were calculated in the validation set with the same formula that was used for calculating the risk-scores of patients in the training set. In the validation set, the distribution of risk-scores and the survival status (Figures 6A,B) had a trend similar to that in the training set between high- and low-risk groups. Also, survival analysis indicated that low-risk patients had significantly favor prognosis than high-risk patients (Figure 6C). ROC curves were used to assess the prognostic value of the risk-scores; the analysis results suggested that risk-scores could accurately predict the survival rate in patients (AUC = 0.776, Figure 6D). The calibration plot in the validation set also showed that the model could accurately predict the 28-day survival (Figure 6E).

## Discussion

ARDS, one of the main critical diseases encountered in intensive care units, is a clinically and pathophysiologically complex syndrome of acute lung inflammation. Despite substantial progress in respiratory support strategies for critically ill patients, including the incorporation of a small tidal volume (21), high positive end-expiratory pressure (22), prone position ventilation (23), lung recruitment (24), use of neuromuscular blockers (25), high-frequency oscillatory ventilation (26, 27), and extracorporeal membrane oxygenation (28, 29), the mortality rate among patients with ARDS remains unacceptably high (30). However, to our knowledge, no study has previously developed a nomogram to predict the prognosis of patients with ARDS.

Herein, we first developed a nomogram using simple and easily available variables to evaluate the 28-day survival probabilities of ARDS patients whose information were extracted from an online database. Thereafter, we tested the performance of the nomogram in training and validation cohorts. Seven risk factors were identified in this model: age, Alb, PLT, PaO_2_/FiO_2_, LDH, CT score, and ARDS etiologies. Additionally, our results showed that PaO_2_/FiO_2_, and CT score could, albeit less accurately, predict the survival probability of ARDS patients compared to our novel model. These results suggest that the nomogram could be used as a cost-effective tool to predict the prognosis of ARDS and assist with clinical decision-making.

In 2012, the Berlin ARDS Society defined the severity of ARDS according to the oxygenation index (5). The oxygenation index (PaO_2_/FiO_2_) was helpful to categorize ARDS patients with different severity, and the mortality was reported to be higher in more severe stages of ARDS (mild, moderate, or severe) (5, 31). However, these severity categories have a low-to-moderate prognostic value to predict respiratory failure (32). Kamo and colleagues (33) reported that the severity stratification of the Berlin ARDS criteria may have a low capacity to differentiate between mild and moderate ARDS. In this study, the results of ROC curve analysis also indicated that the oxygenation index had low prognostic power (AUC, 55.3204%), which was consistent with previous studies.

CT or other lung imaging techniques have been used as diagnostic tools to optimize lung assessment and ventilator management in patients with ARDS; however, it is still controversial whether CT findings can predict ARDS outcomes (34–36). HRCT scores have been reported to correlate with the pathological stage of diffuse alveolar damage (37). Ichikado and colleagues (38) noted that HRCT score was one of the independent predictors of death and ventilator dependency in ARDS patients. Simultaneously, HRCT score was also found to be associated with multiorgan failure and ventilator-associated complications (38). In the present study, to increase model accuracy, HRCT score was incorporated into the nomogram. To evaluate the performance of HRCT score as a prognostic biomarker for the survival of ARDS patients, we performed ROC analysis. Our results showed that the model fit was significantly better than that of the one-factor HRCT model.

APACHE II score can be used as indicators to evaluate the prognosis among critically ill patients; it has been used worldwide to measure ICU performance (39). As APACHE II score included age and other factors in the calculation process, and repeated operations would be generated if the model was built again, APACHE II was not included in the LASSO regression analysis. The APACHE II score is calculated based on acute physiological parameters and chronic health conditions, all of which have significant effects on the predictive prognosis of ICU patients (40). Hwang and colleagues (41) revealed that APACHE II score was a mortality predictor for ARDS patients, but that the accuracy was not high. Lesur and colleagues (42) reported that APACHE II score may be less predictive value when applied for ARDS patients, and that in those patients, it might be less accurate than other indicators, such as age. In the present study, it was also found that the prediction accuracy of this model was better than APACHE II score when compared to the results of precious study (AUC = 0.623) (41).

Certain drugs have also been reported to have the potential to cause ARDS. It has been proved that molecular targeted therapy, such as methotrexate and certain herbal medicines, can cause severe respiratory failure or ARDS (43–45). However, only few studies have focused on the prognostic role of different etiologies of ARDS. In the present study, our results indicated that there is a lower risk of death if ARDS is caused by drugs. However, these discrepancies may be partly related to differences in the dose and duration of drug treatments.

Our study has some limitations. Firstly, the model was developed mainly based on the seven variables. As these factors were unstable throughout the whole follow-up period, which may partly influence the precision of the model. Secondly, only 197 patients were included in this study; further studies with bigger sample sizes are needed. Thirdly, the lack of external validation may limit the extrapolation of the nomogram.

To summarize, we identified eight variables and developed a novel model to predict prognosis in patients with ARDS. These results may help to further improve clinical decision-making and individualized treatment of ARDS patients. Also, this model could distinguish patients with high-risk of ARDS, and further help to perform a careful follow-up among those patients.

## Data Availability Statement

The raw data supporting the conclusions of this article will be made available by the authors, without undue reservation.

## Ethics Statement

Ethical review and approval was not required for the study on human participants in accordance with the local legislation and institutional requirements. Written informed consent for participation was not required for this study in accordance with the national legislation and the institutional requirements.

## Author Contributions

YL and LH designed the study. YL and JL performed the data analysis statistical analysis. YL prepared the manuscript. LH contributed funding for the project. All authors read and approved the final manuscript.

## Conflict of Interest

The authors declare that the research was conducted in the absence of any commercial or financial relationships that could be construed as a potential conflict of interest.

## Abbreviations

Alb: albumin
PLT: platelet count
WBC: white cell count
CRP: C reactive protein
SOFA: sequential organ failure assessment
APACHE: acute physiology and chronic health evaluation
LDH: lactate dehydrogenase
HRCT: high-resolution computed tomography
DARDS: drug-associated ARDS.
